# National and subnational plans for primary prevention and early detection of oral and oropharyngeal cancer: a scoping review

**DOI:** 10.1590/0102-311XEN233923

**Published:** 2025-01-13

**Authors:** Marcia Frias Pinto Marinho, Maria Clara Frias Lobo Marinho, Guido Artemio Marañón-Vásquez, Keith Bullia da Fonseca Simas, Mário José Romañach, Aline Corrêa Abrahão, Maria Augusta Visconti Rocha Pinto, Lucianne Cople Maia de Faria, Michelle Agostini

**Affiliations:** 1 Faculdade de Odontologia, Universidade Federal do Rio de Janeiro, Rio de Janeiro, Brasil.; 2 Faculdade de Odontologia, Universidade do Estado do Rio de Janeiro, Rio de Janeiro, Brasil.

**Keywords:** Health Policy, Mouth Neoplasm, Primary Prevention, Early Detection of Cancer, Risk Factors, Política de Saúde, Neoplasias Bucais, Prevenção Primária, Detecção Precoce de Câncer, Fatores de Risco, Política de Salud, Neoplasias de la Boca, Prevención Primaria, Detección Precoz del Cáncer, Factores de Riesgo

## Abstract

This scoping review maps primary prevention and early detection strategies for oral and oropharyngeal cancer across national cancer plans and noncommunicable disease plans from all World Health Organization Member States. Following PRISMA-ScR guidelines, bibliographic search was performed on key organization websites until March 2023. Of the 194 countries assessed three had subnational plans, resulting in 264 self-governing political entities and similar with revised plans. Among these, 124 (47%) addressed oral and oropharyngeal cancer risk factors and preventive strategies, including 73 national and 51 subnational plans (one from Australia, two from the United Kingdom and 48 from the United States) across 76 (39.2%) countries. Southeast Asia led with 81.8% self-governing political entities mentioning oral and oropharyngeal cancer risk factors and preventive strategies, followed by the Americas (63.5%). Western Pacific and Eastern Mediterranean regions had the lowest coverage with 24.2% and 23.8%, respectively. Tobacco use was the most discussed oral and oropharyngeal cancer risk factor in primary prevention plans (63.7%), followed by HPV infection (54%) and alcohol consumption (35.5%). Opportunistic examination was the most common strategy for early detection, recommended by 29% of self-governing political entities, followed by screening in high-risk individuals (14.5%), self-examination (5.6%), and population-based screening (2.4%). Despite the high oral and oropharyngeal cancer incidence in many countries, most cancer plans only indirectly covered it and showed a great diversity of preventive strategies. Missing data in available documents should not imply an absence of an oral and oropharyngeal cancer policy. Other documents may exist but were not available on the websites, highlighting potential bias.

## Abstract (pt)

Esta revisão de escopo visa mapear estratégias de prevenção primária e detecção precoce de câncer de boca e orofaringe em planos de contenção de câncer e de doenças não transmissíveis de todos os Países Membros da Organização Mundial da Saúde. Seguindo as diretrizes do PRISMA-ScR, foi realizada uma pesquisa nos principais *sites* de organizações-chave até março de 2023. Três dos 194 países avaliados tinham planos subnacionais, resultando em 264 entidades políticas autônomas e similares com planos revisados. Entre estes, 124 (47%) abordaram fatores de risco e estratégias preventivas para o câncer de boca e orofaringe, incluindo 73 planos nacionais e 51 subnacionais (um da Austrália, dois do Reino Unido e 48 dos Estados Unidos) em 76 (39,2%) países. O Sudeste Asiático liderou com 81,8% de entidades políticas autônomas mencionando fatores de risco e estratégias preventivas dos cânceres estudados, seguido pelas Américas (63,5%). As regiões do Pacífico Ocidental e do Mediterrâneo Oriental tiveram as menores coberturas, com 24,2% e 23,8%, respectivamente. O uso de tabaco foi o fator de risco mais discutido para câncer de boca e orofaringe nos planos de prevenção primária (63,7%), seguido pela infecção pelo HPV (54%) e consumo de álcool (35,5%). A estratégia mais comum para detecção precoce foi o exame oportuno, recomendado por 29% das entidades políticas autonomas, seguido de rastreamento de indivíduos de alto risco (14,5%), autoexame (5,6%) e rastreamento de base populacional (2,4%). Apesar da alta incidência de câncer de boca e orofaringe em muitos países, a maioria dos planos oncológicos não cobriam estas condições especificamente, e uma grande diversidade de estratégias preventivas foi observada entre os planos. A falta de informações nos documentos disponíveis não deve implicar a ausência de uma política de câncer de boca e orofaringe; apesar da existência de outros documentos, eles não estavam acessíveis nos sites, destacando possíveis vieses.

## Abstract (es)

Esta revisión de alcance tiene como objetivo mapear estrategias para la prevención primaria y la detección temprana del cáncer de boca y orofaringe en los planes de contención del cáncer y de enfermedades no transmisibles de todos los Países Miembros de la Organización Mundial de la Salud. Siguiendo las directrices del PRISMA-ScR se realizó una búsqueda en las principales páginas *web* de organizaciones clave hasta marzo del 2023. Tres de los 194 países evaluados contaban con planes subnacionales, totalizando 264 entidades políticas autónomas y afines con planes revisados. Entre ellos, 124 (47%) abordaron factores de riesgo y estrategias preventivas para el cáncer de boca y orofaringe, incluidos 73 planes nacionales y 51 subnacionales (uno de Australia, dos del Reino Unido y 48 de Estados Unidos) en 76 (39,2%) países. El Sudeste Asiático lideró el cuadro, sumando el 81,8% de entidades políticas autónomas que mencionaron factores de riesgo y estrategias preventivas para los cánceres estudiados, seguido por las Américas (63,5%). Las regiones del Pacífico Occidental y del Mediterráneo Oriental tuvieron la cobertura más baja, con un 24,2% y un 23,8%, respectivamente. El consumo de tabaco fue el factor de riesgo de cáncer de boca y orofaringe más discutido en los planes de prevención primaria (63,7%), seguido de la infección por HPV (54%) y el consumo de alcohol (35,5%). La estrategia más común para la detección temprana fue el tamizaje oportuno, recomendado por el 29% de las entidades políticas autónomas, seguido del tamizaje de personas de alto riesgo (14,5%), el autoexamen (5,6%) y el tamizaje de base poblacional (2,4%). A pesar de la alta incidencia de cáncer de boca y orofaringe en muchos países, la mayoría de los planes de oncología no cubría específicamente estas afecciones y se observó una amplia diversidad de estrategias preventivas entre los planes. La falta de información en los documentos disponibles no debe implicar la ausencia de una política de cáncer de boca y orofaringe; a pesar de la existencia de otros documentos, no eran accesibles en los sitios web, lo que pone de relieve posibles sesgos.

## Introduction

According to the GLOBOCAN 2020 database from the International Agency for Research on Cancer (IARC), an estimated 500,000 oral and oropharyngeal cancer cases occurred worldwide in 2020. This disease has a high incidence in South and Southeast Asia and the Western Pacific. Yet, countries like Brazil, United States, and several European nations also report expressive numbers ^1^. Oral and oropharyngeal cancer predominantly affects men ^1^ ^,^ ^2^, often leading to late-stage diagnoses, elevated mortality and morbidity rates, high treatment and rehabilitation costs, and significant social burden ^3^ ^,^ ^4^. Control of risk factors and early detection remain the most effective strategies for preventing oral and oropharyngeal cancer and increasing survival rates.

Primary prevention, aimed at averting disease onset, includes public education about risk factors such as discouraging tobacco use, limiting alcohol intake, promoting sun-safe lip protection, advocating for HPV vaccination, and emphasizing the importance of a healthy diet ^2^ ^,^ ^5^ ^,^ ^6^.

Secondary prevention focuses on early diagnosis which is paramount in identifying early-stage oral and oropharyngeal cancer and oral potentially malignant disorders ^7^ ^,^ ^8^. Given its silent onset, many patients only seek professional help when experiencing pain or difficulties with eating, speaking, or swallowing, contributing to delayed oral and oropharyngeal cancer diagnoses ^1^ ^,^ ^2^ ^,^ ^9^ ^,^ ^10^. Visual examination offers a simple, non-invasive, inexpensive, safe, and easily accessible method for detecting suspicious oral lesions, with diagnosis confirmation achieved by incisional biopsy ^2^ ^,^ ^8^. This assessment can be conducted opportunistically during routine dental appointments or as part of a screening program, which can be population-based or geared towards high-risk individuals. The latter, such as tobacco and alcohol users, might not regularly visit the dentist, rendering the opportunistic approach less effective ^3^ ^,^ ^10^. Indeed, visual screening focusing on high-risk individuals ^10^ was associated with a reduction in mortality as demonstrated by the Kerala Oral Cancer Screening Trial in India, the sole randomized study conducted on this matter ^3^ ^,^ ^11^. Unlike breast and cervical cancer, population-based oral cancer screening has not proven to be a fully effective approach ^9^ ^,^ ^12^.

Considering the scarcity of data concerning oral and oropharyngeal cancer prevention, as highlighted by the IARC Perspective on Oral Cancer Prevention ^13^, this scoping review sought to systematically map primary prevention and early detection strategies for oral and oropharyngeal cancer as outlined in the national cancer plans and noncommunicable disease (NCD) plans of all World Health Organization (WHO) Member States, looking for essential differences and possible gaps in prevention efforts.

## Materials and methods

### Protocol and registration

The study protocol was registered in the Open Science Framework (OSF) platform on July 22, 2022 (https://osf.io/89jf5), and available at https://doi.org/10.17605/OSF.IO/Z59BM. This review was conducted and reported following the *Preferred Reporting Items for Systematic Reviews and Meta-Analyses for Scoping Reviews* (PRISMA-ScR).

### Eligibility criteria

Eligibility criteria were established following the Participants-Concept-Context (PCC) framework recommended by the Joanna Briggs Institute (JBI) for scoping reviews ^14^.

Participants: adults;

Concept: cancer plans that addressed primary prevention (including tobacco, alcohol, diet, ultraviolet radiation exposure, and HPV) and secondary prevention (opportunistic examination, population-based screening, screening in high-risk individuals, self-examination, and telemedicine) of oral and oropharyngeal cancer;

Context: WHO Member States.

The main research questions, sub-questions and supplementary data table are intricately linked with the scoping review protocol registered on the OSF.

**Figure 1:**
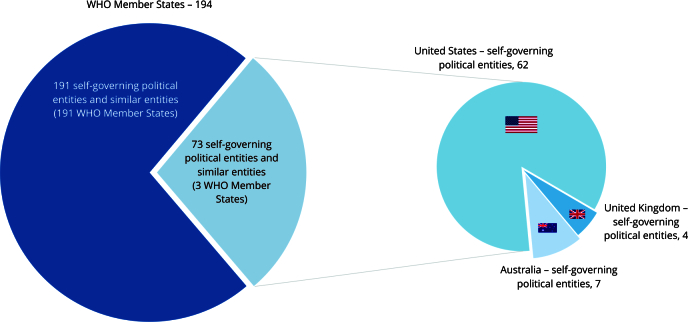
Self-governing political entities and similar entities (n = 264).

Some of the 194 WHO Member States developed subnational plans for their different regions, such as Australia (Western Australia, Northern Territory, Queensland, South Australia, New South Wales, Victoria, and Tasmania), the United Kingdom (England, Wales, Scotland, and Ireland), and the United States with its 62 units (states, districts, territories, and tribes). Thus, this study searched for cancer plans on 264 self-governing political entities or similar bodies (territories, tribes, among others) (Figure 1).

WHO: World Health Organziation.

### Information sources

Websites with potentially relevant documents on efforts against cancer like the International Cancer Control Partnership (ICCP; https://www.iccp-portal.org/), the U.S. National Comprehensive Cancer Control Program (NCCCP; https://www.cdc.gov/cancer/ncccp/index.htm), and the European Partnership for Action Against Cancer (EPAAC; http://www.epaac.eu/) were consulted until March 28, 2023. We set no restrictions on the date, language, or status of the documents.

### Search strategy

Initial searches were conducted on the ICCP and EPAAC websites. Self-governing political entities and similar entities were selected in the “national plans” section and their most recent cancer plans and NCD plans (only available on the ICCP) were extracted. Searches on the NCCCP website were performed individually for each state, territory, or tribe, resulting in the selection of the most recent document available. Documentary search was first conducted in English and modified to match the language of the region of interest. Translation was achieved with help of the Google Translate application (https://translate.google.com). We evaluated plans in various languages, including Spanish (e.g., Chile, Cuba, Guatemala, Panama), French (e.g., Ivory Coast, Mauritania, Senegal, Belgium, Switzerland), German (e.g., Austria), Bosnian (Bosnia and Herzegovina), Portuguese (e.g., Brazil, Cape Verde, Portugal), Czech (Czech Republic), Greek and Japanese.

### Selection process

Selection was performed by two researchers (M.F.P.M. and M.C.F.L.M.). Both searched for plans on the websites, determined which were potentially eligible according to the eligibility criteria, and assessed the texts in full. Most documents were in English and for those not available in English, Google Translator application aided the translation. Some plans from the same self-governing political entity were excluded due to duplicity or because they addresses specific types of cancer (e.g., breast, cervix). When more than one plan was available for the same self-governing political entity or similar entity, selection considered only the most recent. Documents were then excluded if they failed to address the following terms related to cancer: “oral”, “oropharyngeal”, “oropharynx”, “pharyngeal”, “pharynx”, “mouth”, “lip”, “head and neck”, “oral squamous cell carcinoma”, “oropharyngeal squamous cell carcinoma”, or “throat”. Of the remaining documents, only those that discussed risk factors and oral and oropharyngeal cancer prevention strategies were included. A consensus meeting was held between the researchers. Disagreements were resolved by a third reviewer (M.A.).

### Data extraction

A draft-charting form developed in Microsoft Excel spreadsheets (https://products.office.com/) was used to determine which data to extract. Two researchers (M.F.P.M. and M.C.F.L.M.) independently entered the following information: document characteristics (title, type, year of publication, expiration date), access link, primary prevention strategies for oral and oropharyngeal cancer (e.g., tobacco control, limiting alcohol consumption, HPV vaccination, diet, and sun exposure protection), and secondary prevention strategies (e.g., population-based screening, screening of high-risk individuals, opportunistic screening, self-exam recommendation, and telemedicine as an aid to diagnosis). In case of disagreement, a third reviewer (M.A.) was consulted to reach a consensus. Additional strategies to reduce the oral and oropharyngeal cancer burden were also extracted.

### Synthesis of results

Results were categorized according to the main public strategies. A check table featuring self-governing political entities or similar entities grouped by WHO regions was created. Plans were presented on the rows. Topics related to the sub-questions were represented in columns which enabled identifying strategies, concept reviews, and additional information are available at: https://doi.org/10.17605/OSF.IO/Z59BM.

## Results

### Selected plans

**Figure 2:**
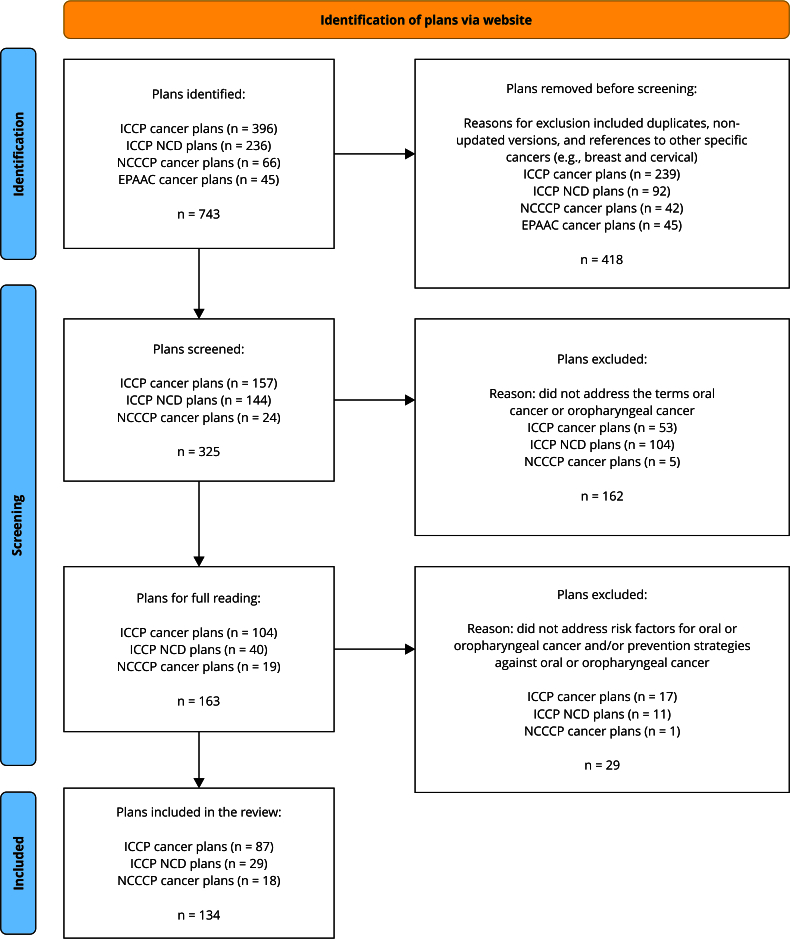
Flowchart of the data collection process.

EPAAC: European Partnership for Action Against Cancer; ICCP: International Cancer Control Partnership; NCCCP: U.S. National Comprehensive Cancer Control Program; NCD: noncommunicable disease.

Website search identified a total of 743 documents. Of the 264 self-governing political entities and similar entities, some had a cancer plan whereas others presented only an NCD plan, some had both and others had none. After applying the selection criteria, 325 plans remained. Of these, 162 were excluded for not addressing the established cancer-related terms. The remaining 163 plans had their full text examined in more detail. Plans that failed to discuss oral and oropharyngeal cancer risk factors or did not list primary or secondary prevention strategies for oral and oropharyngeal cancer (n = 29) were removed. Finally, 134 plans from 124 self-governing political entities and similar entities were included in the study (Figure 2). Thus, only 124 (47%) of the 264 self-governing political entities and similar entities addressed risk factors and preventive strategies for oral and oropharyngeal cancer, including 73 national plans and 51 subnational plans (one from Australia, two from the United Kingdom and 48 from the United States) across 76 (39.2%) WHO Member States. Southeast Asia had the highest percentage of self-governing political entities and similar entities with oral and oropharyngeal cancer strategies (81.8%), followed by America (63.5%). Western Pacific (24.2%) and the Eastern Mediterranean region (23.8%) had the lowest coverage.

### Characteristics of selected plans

Of the 134 documents included, 116 were retrieved from the ICCP website (87 cancer plans and 29 NCD plans), and 18 cancer plans were obtained from the NCCCP website. Of the 124 self-governing political entities and similar entities whose plans were reviewed, 62 (50%) had updated documents and 54 (43.55%) had outdated documents, defined as those with expiration dates up to and including 2023. Moreover, 8 (6.45%) featured only the publication dates. Netherlands, Niger, Palau, Indiana and the American Indian Cancer Foundation (both from the United States) presented plans with the shortest period (two years). Finland and Saudi Arabia had plans with the longest expiration date (11 years). Most self-governing political entities and similar entities presented plans with an expiration date of four years (38%). Oregon’s plan (United States) went the longest without updating (last update in 2005). Ireland had the document with the longest expiration date (2022-2032). Summary of the primary data and the link to the documents are presented in supplementary data register: https://osf.io/z59bm; protocol: https://osf.io/89jf5/.

### Oral and oropharyngeal cancer preventive strategies in the plans

Preventive strategies were categorized into primary and secondary prevention (early detection) actions for oral and oropharyngeal cancer: 84.7% of the plans addressed risk factors and oral and oropharyngeal cancer-related strategies to combat them, and 46% recommended strategies for early detection (Table 1).

**Table 1:** Primary and secondary prevention strategies for oral or oropharyngeal cancer by self-governing political entities and similar entities (grouped according to World Health Organization regions).

Assessed variables	Self-governing political entities and similar entities [n (%)]						
	Africa	America	Europe	Eastern Mediterranean	Southeast Asia	Western Pacific	Total
Prevention strategy for oral or oropharyngeal cancer							
With oral or oropharyngeal cancer risk factors and preventive strategies against oral or oropharyngeal cancer	19 (40.4)	61 (63.5)	22 (39.3)	5 (23.8)	9 (81.8)	8 (24.2)	124 (47.0)
Without oral or oropharyngeal cancer risk factors and preventive strategies against oral or oropharyngeal cancer	28 (59.6)	35 (36.5)	34 (60.7)	16 (76.2)	2 (18.2)	25 (75.8)	140 (53.0)
Oral or oropharyngeal cancer risk factors and preventive strategies for oral or oropharyngeal cancer *							
Primary prevention	11 (57.9)	54 (88.5)	22 (100.0)	4 (80.0)	7 (77.8)	7 (87.5)	105 (84.7)
Tobacco control	8 (42.1)	40 (65.6)	14 (63.6)	3 (60.0)	7 (77.8)	7 (87.5)	79 (63.7)
Limit alcohol consumption	7 (36.8)	17 (27.9)	13 (59.1)	0 (0.0)	4 (44.4)	3 (37.5)	44 (35.5)
HPV vacine	7 (36.8)	38 (62.3)	15 (68.2)	1 (20.0)	4 (44.4)	2 (25.0)	67 (54)
Diet control	2 (10.5)	6 (9.8)	2 (9.1)	0 (0.0)	2 (22.2)	1 (12.5)	13 (10.5)
Sun exposure control	0 (0.0)	2 (3.3)	0 (0.0)	0 (0.0)	0 (0.0)	0 (0.0)	2 (1.6)
Secondary prevention	9 (47.4)	27 (44.3)	5 (22.7)	2 (40.0)	9 (100.0)	5 (62.5)	57 (46.0)
Opportunistic examination	6 (31.6)	20 (32.8)	4 (18.2)	2 (40.0)	3 (33.3)	1 (12.5)	36 (29.0)
Population-based screening	0 (0.0)	1 (1.6)	0 (0.0)	0 (0.0)	2 (22.2)	0 (0.0)	3 (2.4)
Screening of high-risk individuals	3 (15.8)	6 (9.8)	1 (4.5)	0 (0.0)	4 (44.4)	4 (50.0)	18 (14.5)
Self-examination	0 (0.0)	3 (4.9)	0 (0.0)	1 (20.0)	3 (33.3)	0 (0.0)	7 (5.6)

* Percentages were calculated based on the total number of self-governing political entities and similar entities (n = 124) with oral or oropharyngeal cancer risk factor and preventive strategies for oral or oropharyngeal cancer.

### Primary prevention for oral and oropharyngeal cancer

**Figure 3:**
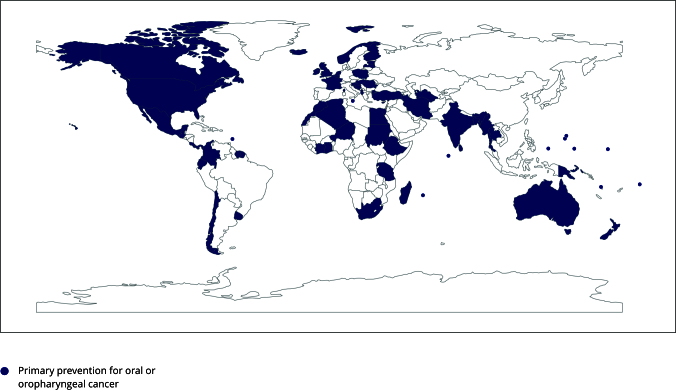
World Health Organization (WHO) Member States with cancer plans that include information on risk factors and primary preventive strategies for oral or oropharyngeal cancer.

Source: prepared by the authors, using Python (https://www.python.org/) within the Jupyter Notebook (https://jupyter.org/) environment employing the *GeoPandas* and *Matplotlib* libraries. Note: for WHO-registered countries, the noncolored ones failed to provide information about primary prevention on the websites of the International Cancer Control Partnership (ICCP; https://www.iccp-portal.org/), the U.S. National Comprehensive Cancer Control Program (NCCCP; https://www.cdc.gov/cancer/ncccp/index.htm), and the European Partnership for Action Against Cancer (EPAAC; http://www.epaac.eu/). In the United States, most states reported primary prevention strategies for oral or oropharyngeal cancer. In Australia, only the Victorian region’s plan included information on primary prevention for oral or oropharyngeal cancer, whereas in the United Kingdom, England and Northern Ireland outlined primary prevention strategies for oral or oropharyngeal cancer.

Searching risk factors and primary prevention strategies for oral and oropharyngeal cancer revealed that 84.7% of the surveyed self-governing political entities and similar entities addressed these aspects, but not all of them covered all oral and oropharyngeal cancer risk factors comprehensively (Table 1). Of these, 63.7% highlighted the association between tobacco use and oral and oropharyngeal cancer, whereas 35.5% emphasized alcohol intake. HPV vaccination to prevent oropharyngeal cancer was mentioned in 54%. A healthy diet was cited as a protective factor for oral and oropharyngeal cancer by 10.5% of self-governing political entities and similar entities, whereas only U.S. states of Illinois and Oregon (1.6%) listed sun exposure as a specific risk factor for lip cancer (Table 1). Notably, 20% of the self-governing political entities and similar entities linked oral and oropharyngeal cancer only to tobacco, 2% to alcohol consumption, and 22% exclusively associated it with HPV infection. In most plans, association with HPV was related to oropharyngeal (throat) cancer, but some documents linked it to head and neck cancer and mouth cancer. Considering all risk factors, 9.5% related oral and oropharyngeal cancer to tobacco and alcohol use and 19.1% associated it to the three most cited risk factors, i.e., tobacco, alcohol, and HPV. Only Illinois linked oral and oropharyngeal cancer to the five leading risk factor: tobacco, alcohol, HPV, diet, and sun exposure (Table 2). Other associations were found in 27.6% of self-governing political entities and similar entities with oral and oropharyngeal cancer risk factors and prevention strategies in their plans. Figure 3 highlights WHO Member States with cancer plans that include information on risk factors and primary prevention strategies for oral and oropharyngeal cancer. The diverse initiatives outlined in these documents aimed at preventing oral and oropharyngeal cancer by minimizing exposure to recognized risk factors were summarized and categorized into three levels: policy-based (regulatory), system-focused (community), and awareness-driven (educational systems) (Box 1).

**Table 2:** Oral or oropharyngeal cancer risk factors most frequently addressed in the plans of self-governing political entities and similar entities (grouped according to World Health Organization regions).

Assessed variables	Risk factors most associated with oral or oropharyngeal cancer [n (%)]						
	Africa	America	Europe	Eastern Mediterranean	Southeast Asia	Western Pacific	Total
Primary prevention	11	54	22	4	7	7	105
Only tobacco-related	1 (9.1)	11 (20.4)	2 (9.1)	3 (75.0)	1 (14.3)	3 (42.8)	21 (20.0)
Only alcohol-related	0 (0.0)	0 (0.0)	2 (9.1)	0 (0.0)	0 (0.0)	0 (0.0)	2 (1.9)
Only HPV-related	3 (27.3)	12 (22.2)	6 (27.3)	1 (25.0)	0 (0.0)	0 (0.0)	22 (21.0)
Related to tobacco and alcohol	1 (9.1)	4 (7.4)	2 (9.1)	0 (0.0)	2 (28.6)	1 (14.3)	10 (9.5)
Related to tobacco, alcohol and HPV	4 (36.3)	7 (13.0)	7 (31.8)	0 (0.0)	1 (14.3)	1 (14.3)	20 (19.1)
Related to tobacco, alcohol, HPV, diet and sun exposure of the lips	0 (0.0)	1 (1.8)	0 (0.0)	0 (0.0)	0 (0.0)	0 (0.0)	1 (0.9)
Other associations	2 (18.2)	19 (35.2)	3 (13.6)	0 (0.0)	3 (42.8)	2 (28.6)	29 (27.6)

Note: percentages were calculated based on the total number of self-governing political entities and similar entities (n = 124) from 76 World Health Organization countries with oral or oropharyngeal cancer risk factors and preventive strategies for oral or oropharyngeal cancer.

**Box 1:** Primary prevention strategies to reduce the risk of oral and oropharyngeal cancer.

GOAL	LEVEL	PRIORITY STRATEGIES
Reduce incidence rates of tobacco-related cancers	Regulatory 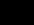	Update national tobacco control policy
		Increased tax on tobacco products
		Smoke-free environments law
		Prohibiting sales to under-18-year-olds
		Raise the minimum legal sale age of tobacco products, including electronic cigarettes and nicotine delivery devices, to 21 years old
		Earmark a portion of tobacco taxes for tobacco control fund and/or enforcement efforts
		Increase funding for tobacco product cessation programs
		Clear and explicit health warnings on cigarette packs
		Ban all advertising, promotion and sponsorship of tobacco products
		Advocate laws requiring tobacco product retailers to be licensed
		Reducing the total number of retail tobacco licenses
		Prohibited to sell tobacco and related tobacco products online, and to sell tobacco and related tobacco through vending machines
		Develop a tracking system for the sale of electronic cigarettes/devices to youth under the age of 18
		Ban on sale of single sticks, etc.
		Ensure that effective measures are taken to minimize the entry of illicit and therefore cheaper tobacco products
		Pass legislation to ban smoking in vehicles carrying children
		Develop a standard operating procedure for imposition of penalties in tobacco rule violation
		Guarantee the execution of 100% of the resources coming from the account of fines for sanctions for noncompliance with tobacco law, in projects for the prevention of consumption and exposure to tobacco smoke and its derivatives
		Prohibited to show persons smoking or consuming the products referred to on television
		Having a record and reports of bans on all scenes of tobacco use
		Creation and implementation of an anti-smoking policy in mental health institutions
		Conduct regular quarterly surveys every year of existing local and jurisdiction-wide tobacco-related policies and laws utilizing the tracking system
		Require that inexpensive cigars be sold in multiunit packs with a hard price floor
		Establish tobacco-free outdoor areas including major employers, parks, playgrounds and beaches
		Increase border control & in-country spot-checks
		Develop and produce captions and signs for non-smoking areas
		Implement or enforce smoke-, tobacco- and vape-free laws and regulations for hospitals, schools, colleges and universities
		Work with elected officials to create a minimum distance that smoking can occur around government buildings
		Levy higher taxes on areca nut imports and sales
		Health warnings on areca nuts packaging identifying this as a carcinogen
		Develop and disseminate model tobacco-free policies that prohibit the use of nicotine delivery systems (e.g., e-cigarettes) and electronic smoking devices
		Legislate to ensure vaping is regulated appropriately: New Zealand urgently needs legislation to regulate vaping products that supports smokers to switch to a less harmful alternative while protecting children and youth from access to and use of these products
		Require that electronic nicotine delivery devices, including e-cigarettes, be held to the same regulatory, advertising, promotion and sponsorship standards as all other tobacco and nicotine products
		Prohibited to smoke and consume tobacco and related products, including smokeless tobacco products, electronic cigarettes and herbal products for smoking, during public performances
		Prohibit sale of flavored tobacco products including menthol in cigarettes and all flavored electronic cigarette products
		Protecting public health policies related to tobacco control against commercial interests and other vested interests of the tobacco industry
		Responsibility of the Public Health Ministry for carrying out targeted inspections to emsure compliance with the “tobacco” laws
	Community 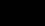	Activities in support of the implementation of the law that prevents passive smoking in public environments (awareness campaigns, administrative measures)
		Public campaign for the prevention of smoking, especially in groups that are assessed as dangerous in growth as women and youth
		Campaign in schools and workplaces
		Campaign for Tobacco Free Homes
		Designing and implementing personalized communication campaigns across different media platforms to achieve the following goals: Enhancing awareness about different cancer risk factors; Disseminating age-appropriate messages to ensure effective communication and engagement; Encouraging smoking cessation by promoting quitting attempts and increasing successful quitting rates among smokers, with a specific focus on adults from low-socioeconomic backgrounds
		Develop and implement a communication program on the benefits of physical activity as a means of combating smoking
		Promoting healthy lifestyle models in social networks (including Tik-Tok, Facebook, Instagram) by collaborating with influential people among youth and children, such as athletes or famous artists
		Organize activities for World No Tobacco Day
		Encourage, increase, and review research to determine effects of current and emerging risk factors
		Supporting evidence-based tobacco prevention and cessation programs that target both youth and adults
		Develop specific betel nut control initiatives addressing adults and youth to assure comprehensive, culturally appropriate media messages reach the intended audience
		Disseminate the latest tobacco prevention, cessation and control research findings as available
		Opinion leaders’ workshop on tobacco and cancer programs
		Conduct *Global Youth Tobacco Survey* and *School Health Survey*
		Monitor trends in data related to cancer risk, including trends in relative cancer risk among priority populations
		Assess the economic and epidemiological impact of tobacco consumption
		Publishing and regularly updating (annual) cancer statistics to create awareness among public and policymakers of statistics regarding the most prevalent cancers, risk factors linked to the development of these cancers, and financial burden of care
		Promote the use of evidence-based strategies for preventing youth from initiating tobacco
		Reduce the attractiveness of tobacco products by putting up dissuasive posters and panels for the attention of patients and their companions
		Routinely conduct assessments that highlight the actual usage of emerging products
		Creation of a phone application based on scientific research to quit smoking
		Create a messaging tool centered around “Don’t start smoking at all” as the main message
		Develop tools to monitor data on substance abuse
		Develop a tracking system for the sale of electronic cigarettes/devices to youth under the age of 18
		Develop a national communication program on “smoking, an avoidable risk factor for cancer” in health establishments
		Implement the clinical practice guideline for cessation tobacco and nicotine replacement therapy
		Introduce animations on “tobacco and cancer” in the consultation rooms
		Train health personnel for the implementation of the brief counseling as a tool for the identification of risks and cessation of the consumption of tobacco and its derivatives
		Train health personnel in current regulations related to the control of tobacco use and its derivatives
		Develop training materials and conduct training of trainers on tobacco enforcement
		Develop and implement educational strategies for health service professionals, as a complement to mass communication and community interventions in the issues of control of consumption and exposure to secondhand smoke tobacco and its derivatives
		Capacity building in the health system to help those who wants to quit smoking
		Conduct community needs assessments on an ongoing basis to assess barriers and infrastructure needs for accessing evidence-based tobacco cessation services
		Reduce barriers to initial and continued access to evidence-based tobacco cessation programs
		Promote tobacco cessation referrals and interventions for cancer patients who continue to use tobacco at any stage during and after cancer diagnosis
		Promote implementation of electronic referral to cessation services within electronic health records to among health care facilities, cancer centers and health care associations
		Encourage healthcare providers to ask their patients about secondhand smoke and electronic cigarette aerosol, and provide those patients with evidence-based strategies to reduce such exposure
		Encourage health care workers to ask parents of young children and youth if they use tobacco and if tobacco is used in their homes, to determine their readiness to quit and advise them accordingly
		Continue to augment capacity and quality of services supporting smoking cessation
		Increase the number of health care providers and systems that fully integrate tobacco use treatment into the clinical and community health workflow
		Revise medical catalogues to include drugs for prevention of substance abuse and/or treatment of substance use disorders
		Create specialized tobacco clinics by level of intervention
		Make available and accessible the therapeutic means of assistance to weaning
		Deny a smoking cessation service by level of intervention
		Smoking recognized in the customer’s health report and explain the risks of smoking
		Increase the availability of tobacco use cessation services for individuals affected by tobacco-related disparities, such as LGBTQ communities, low socioeconomic status individuals and adults with depressive disorders
		Increase screening of youth for nicotine dependence and tobacco/ENDS/Juul use and increase youth-specific cessation resources
		Expand the adoption of the community health worker model to connect current tobacco users to and/or provide cessation services and education
		Implement health systems change strategies to increase access to and use of evidence-based cessation services
		Implement community outreach and education programs designed to reduce all forms of tobacco use
		Expand access to and use of tobacco cessation services and treatment
		Enhance the quality of capacity development material for training purposes
		Strengthen tobacco monitoring and surveillance
		Inter-ministerial collaboration and local coalitions to promote the Quitline and local cessation efforts
		Partner with the addictions, HIV and STD treatment programs to increase awareness of oral and oropharyngeal cancer risk factors, among high-risk populations
		Partner with other chronic disease programs who share risk factors to maximize effectiveness of resources in addressing reduction of oral cancer mortality rates which include: sponsoring oral cancer continuing education for dental providers in conjunction with the cancer control partnership, thus stressing the importance of cancer screening and early detection
		Sponsoring oral cancer continuing education for dental providers in conjunction with tobacco intervention programs
		Partner with other stakeholders to increase awareness regarding the relationship of oral and oropharyngeal cancer to HPV
		Identifying and education of NGOs dealing with tobacco control
		Implementation of projects in the field of tobacco control with the participation of NGOs
		Increase border control & in-country spot-checks
		Develop a pilot communication program in companies on “tobacco, an avoidable risk factor for cancer” in companies
		Develop a pilot communication program in sports structures on “tobacco, an avoidable risk factor for cancer”
	Education systems 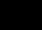	Develop national tobacco awareness programs
		Develop and implement mass media (TV, radio, print, and social media) and campaigns about the necessity and opportunities for tobacco cessation, especially targeting youth with societal pressure on the media
		Establishing a website for sharing available information, educational and communication materials on a permanent basis through a variety of media platforms used by both health professionals and the public and other means of health promotion
		Design, reproduce and disseminate informative and didactic materials to prevent smoking
		Increase the number of health education materials that are presented in culturally appropriate ways
		To increase direct public education to populations at high risk for oral and oropharyngeal cancer with national, state, and local oral and oropharyngeal cancer awareness and education campaigns
		Conduct public educational campaign to support legislation regarding packaging and labeling of tobacco products
		Informing and educating the public, both health professionals and the mass media, to adopt a healthy lifestyle and avoid exposure to risk factors
		Set up an education program targeting youth, particularly vulnerable groups among them
		Educate and inform youth about e-cigarette use to reduce use
		Create videos for prevention focus areas to play in hospital and clinic waiting areas
		Conduct an effective education and awareness campaign about the dangers of secondhand smoke
		Explore and promote telehealth options for tobacco cessation
		Introduce awareness sessions and animations on “tobacco and cancer” in the workplace
		Develop a national communication program on smoking in schools and universities
		Introduce awareness sessions on “tobacco as a risk factor” for teachers and supervisors (harms of tobacco, harms of exposure to tobacco smoke for nonsmokers)
		Increase awareness among education and community officials of the benefits of creating tobacco-free environments for youth
		Incorporating age-appropriate information on cancer prevention and cancer in school curriculums
		Develop a positive attitude about non-smoking among younger generations, and secure a positive environment
		Create a label “School without tobacco”, “University without tobacco”
		Create a “Tobacco-free hospitals“ label
		Create a “Tobacco Free Company“ label
		Create a “Tobacco-free stadium” label
		Develop, distribute, and popularize a national smoking cessation guide for use by health professionals
		Educate policymakers, healthcare professionals, allied health workers, and the public about the need for tobacco and smoke-free environments
		Educate and inform on the public health benefits of raising the tobacco tax
		Set up an education program targeting youth, particularly vulnerable groups among them
		Train health workers, social workers, and academia on the management of substance abuse dependence and addiction
		Coordinate continuing education programs for medical and dental professionals on the primary prevention and early detection of oral and oropharyngeal cancer
		Educate health care providers about evidence-based clinical practice guidelines for tobacco cessation interventions and methods for assisting quit attempts by offering free courses
		Educate physicians and other health care providers about the “5 A’s” for reducing tobacco use
		Enable physicians and nurses to treat tobacco addiction
		Train health personnel for the implementation of the brief counseling as a tool for the identification of risks and cessation of the consumption of tobacco and its derivatives
		Train health personnel in current regulations related to the control of tobacco use and its derivatives
		Educate providers about skin, oral, and prostate cancer screenings, including best practices, benefits and harms, and screening limitations
		Educate patients on how to perform self-screening using a mirror for oral and skin cancer, especially for high-risk patients
		Establish public education campaign that stresses the addictive and carcinogenic nature (oral or oropharyngeal cancer risk) of betel nut use and doma use
		Educate the public on eliminating contrary beliefs that chewing areca nuts promotes oral health and introduce alternative spices for chewing, such as cinnamon and cardamom pods, which would have fewer adverse impacts on oral health
Reduce the incidence rates of cancers related to alcohol intake	Regulatory 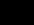	Establishment of the national policy on alcohol consumption
		Law prohibiting the promotion and sponsorship of alcoholic beverages
		Law prohibiting the sale of alcoholic beverages to minors and sales near schools and places of concentration of youth: verification of compliance
		Law to regulate the time or space of the advertisement to warn of the damages of its consumption to health, with restricted broadcast hours and spaces
		Laws to reduce the association between drinking and driving
		Increase taxation on alcohol products
		Limit alcohol advertisements in public locations such as near schools and on public transportation where youth ages 21 years and younger are exposed to marketing
		Promote implementation of legislation on production and consumption of alcohol
		Promote compliance with existing regulations and strengthen sanitary control over alcohol
		Promote healthy warnings on alcohol beverages about the relationship between alcohol and cancer
		Limiting the retail sale of medium-strength beer
		Reduction of beer alcoholic content
		Effective warning labels
		Review existing legislations on alcohol control
		Pursue the earmarking of taxes and licensing fees received on alcohol to go towards alcohol control programs
		Promote responsible beverage service training to store managers for liquor licenses and their employees to improve knowledge and skills on when and how to check customer identification, how to spot fake identification, and how to avoid selling alcohol to intoxicated people
		Engage employers to adopt cancer control plans and to adopt health improvement policies
	Community 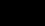	Strengthening intersectoral collaboration with the national agency leading the National Alcohol Policy
		Conduct advocacy meetings for political leadership and policymakers to support the implementation of alcohol policy as a strategy for the prevention and control of cancer
		Develop clinical guidelines in management programs for alcohol dependency in primary care
		Dietary guidelines recommend that those who drink alcohol do so moderately, defined as one drink per day for women and up to two drinks per day for men
		Integrate information on the harmful use of alcohol into routine health education
		Programs focus primarily on reducing the alcohol consumption of children and adolescents and on establishing attitudes of responsible moderation, with a primary focus on protecting at-risk groups
		To include the harmful use of alcohol consumption in all NCD health promotion and campaigns
		To conduct aggressive sensitization campaigns against alcohol, use all year round
		Introduce alcohol and drug tests and management
		Improvement of the system of data collection, monitoring, and reporting related to the use of alcohol among the population
		Among persons meeting the diagnostic criteria for alcohol dependence, promote the use of alcohol misuse screening and brief behavioral counseling interventions via traditional (face-to-face) or electronic means and referrals to specialty treatment
		Promote alcohol behavioral counseling referrals and interventions for cancer patients who continue to use alcohol at any stage during and after cancer diagnosis
		Encourage community coalitions that build partnerships between schools, faith-based organizations, law enforcement, healthcare, and public health agencies to reduce all alcohol consumption among underage youth
		Cooperation with the mass media on the topic of harmful effects of alcohol
		To ensure a positive trend occurs among youth by enhancing in particular children and youth to rely on alcohol advertising controls
		Collaborate with schools and children to identify and implement positive alternative activities that help to prevent alcohol abuse on weekends
		Identify and collaborate with organizations that provide alcohol training and materials targeted at youth
		Identify the group of children with minimal supervision at home or have parents who drink to excess and may therefore be at high risk for problem drinking themselves
		Increase knowledge of why, when, and how to check identification for alcohol purchases by distributing guides to retailers with new liquor licenses
		Evaluate the services for the care of people in the process of alcoholism rehabilitation, and promote the entire population's access to these services fostering greater recognition of alcohol-related harms at the local level and promoting effective and cost-effective responses appropriate to local determinants of harmful use of alcohol and related problems
		Working with employers to promote the introduction of alcohol policies within workplaces with the aim of promoting more awareness, early intervention, and support for employees
		Training of other stakeholders in counseling and rehabilitation of alcohol abuse
		Collaborate with institutes of higher education to support campus safety programs to reduce binge drinking
		Conducting research in the field of nutrition (epidemiological, behavioral, sociological)
	Education system 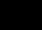	Raise awareness of the harmful effects of alcohol and its relationship with cancer
		Increase parent and youth awareness of alcohol safety and prevention measures using messaging on social media at times youth are known to be at risk for consuming alcohol
		Incorporate awareness messages and information on the risks of alcohol consumption into the school health program
		Introduce the social and health hazards of alcohol use in teacher training modules
		Further implementation of school-based programs that have been shown to be effective
		Promote, coordinate, and evaluate the implementation of information, education, and communication strategies to reduce alcohol consumption, taking into account social participation
		Conduct regular information sharing with the community using preorganized messages
		Designing and implementing communication campaigns that are tailored to different media and audiences with the aim of raising awareness on different risk factors for cancer
		Engage youth in program to promote awareness of the dangers and illegality of purchasing alcohol for underage youth in convenience stores and supermarkets
		Planning training for health care providers in primary care and emergency services to identify persons with hazardous and harmful patterns of alcohol consumption by using tools such as the *Alcohol Use Disorders Identification Test* promoted by WHO
		Promote healthcare provider awareness and use of alcohol screening and brief behavioral counseling interventions to facilitate delivery of personalized feedback about the risks and consequences of excessive drinking
		Provide personalized feedback about the risks and consequences of excessive drinking through the use of electronic screening and behavioral counseling interventions in healthcare settings, schools, and emergency rooms
		Disseminate patient educational materials (print or online) on the harmful effects of alcohol to healthcare providers
Increase HPV vaccines to reduce the incidence of cancer	Regulatory 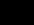	Expanded program with HPV vaccination
		Policymakers should conduct an assessment of current of the topic
		Report immunization status of students to include data for HPV vaccination in addition to the currently required vaccines
		Expand community health worker model by healthcare organizations to promote HPV vaccine messaging
		Coverage of HPV vaccinations to adults
		Issue a Cancer Vaccine Report Card with a focus on cancer
	Community 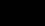	Guidance for effective provider recommendation
		Expand HPV vaccination coverage for target groups
		Expand HPV vaccination coverage for men between the ages of 16 and 26 who have sex with men
		Increase vaccinations to those who miss HPV vaccination
		Reduce missed clinical opportunities to recommend and administer the HPV vaccine
		Scheduling the next appointment, the same day as the first dose is given
		Implementing electronic health record tools that help providers identify patients in need of vaccination
		Implementing patient reminder-recall systems in healthcare to increase the use of the HPV vaccines
		HPV vaccination campaign
		Support HPV vaccination programs in schools
		Collaborate with university-based clinics to offer the HPV vaccine in university-based clinics
		Implement mandatory reporting to a fully robust immunization registry
		Reduce the disparity between boys and girls being up to date on HPV vaccination
		Monitoring HPV vaccination rates
	Education system 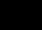	Provide community-wide education on the prevention of sexually transmitted infections
		Increase public awareness of the HPV vaccine proven to reduce the risk of cancer
		Utilize small media (eg, social media, blogs) to increase public awareness
		Educate parents and guardians about the availability and importance of HPV vaccination for adolescents, with a focus on cancer prevention
		Provide education in schools about the prevention and effectiveness of the HPV vaccination
		Offer HPV vaccine continuing education for health care providers
		Increase the number of clinicians strongly recommending the HPV vaccine at the same time they administer meningococcal conjugate and tetanus diphtheria-acellular pertussis vaccines
		Development of educational material
		Share social media content on positive patient experiences and current research
		Deploy co-branded public awareness messaging with prevention partners
		HPV awareness campaign
Reduce the incidence of cancers related to nutrition	Regulatory 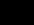	Improve access to acceptable and affordable healthy foods - promoting fruits and vegetables
		Law for the regulation of school feeding
		Law to establish a food labeling system that allows consumers to determine the content and nutritional value of food products
		Law to prohibit the use of trans fats in processed food products and restaurants
		Develop a tax regime for unhealthy foods
		Regulate the importing and advertising of unhealthy foods
		Regulate sales of food through increased taxes
		Consider the earmarking of taxes and licensing fees received on foods to go towards NCD control programs
		Incentives to farmers for the cultivation of fruit and vegetables
		Reduce the promotion of unhealthy foods choice to children
		Engage employers to adopt cancer control plans and to adopt health improvement policies
		Integrate healthy living principles in programs and policymaking
		Implement school policies: prohibit advertising of unhealthy foods and promote healthy foods in schools, including those sold and served within school meal programs
		Connect food-insecure populations to financial assistance programs that make healthy foods more affordable
		Support access to the emergency food system (e.g., food pantries) for people experiencing urgent food needs
	Community 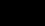	Promote the consumption of fruits and vegetables in schools
		Implement community gardens in schools and make them trendy
		Limit access to sugary drinks in schools, places of work, and healthcare settings
		Promote agreements between schools, communities, parks and recreation, state and local governments, and other groups to increase physical activity opportunities in the community
		Develop behavioral impact activities for the prevention of modifiable risk factors and the adoption of healthy lifestyles in the community, schools, and workplace
		Guidelines with the principle of healthy nutrition based on food diversity, food groups, the amounts consumed daily, and portion sizes according to age and gender
		Health promoting protocol that supports healthy living behaviors in adults
		Engage community health workers to provide education for those at high risk
		Deliver patient education materials (print and online)
		Reducing the use of excessive salt and sugar in places where mass feeding is provided, such as restaurants
		Offering information on quality components of various meals as well as teaching meal planning skills
		Make vegetables trendy through influencer marketing and social media
		Support healthy community design initiatives, such as increasing opportunities for physical activity, to make it easier for people to live healthy lives
		Support worksites in developing policies and programs to promote healthy behaviors
		Research into emerging nutrition issues
		Campaign on obesity
		Increase access to and availability of healthy food choices in schools, workplaces, and communities
		Community campaigns to promote healthy food and beverage choices
		Support existing programs focused on increasing fruit and vegetable intake
		Use essential sources of information, such as the Internet and telephone, to support the services
	Education system 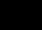	Raising awareness for healthy food choices
		Campaign to raise awareness of healthy food choices
		Public awareness of unhealthy food as a cancer risk factor
		Teach the food guidelines in pre- and primary schools
		Implement nutrition education programming in the community as well as the purchasing e preparations of fruits and vegetables
		Program for healthcare professionals’ education on healthy nutrition
Reduce the incidence of cancers related to sun exposure	Education system 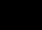	Avoid excess sun exposure
		Reduce expose to natural and artificial ultraviolet radiation

ENDS: electronic nicotine delivery systems; NCD: noncommunicable disease; NGO: nongovernmental organization; STD: sexually transmitted disease; WHO: World Health Organization.

### Secondary prevention for oral and oropharyngeal cancer

**Figure 4:**
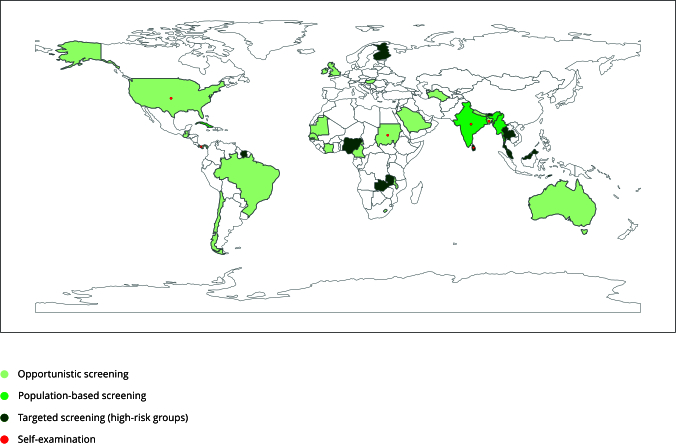
Main strategies of cancer plans for early detection of oral or oropharyngeal cancer.

Source: prepared by the authors, using Python (https://www.python.org/) within the Jupyter Notebook (https://jupyter.org/) environment employing the *GeoPandas* and *Matplotlib* libraries. Note: for WHO-registered countries, the noncolored ones did not have a plan for secondary prevention of oral cancer on the websites of the International Cancer Control Partnership (ICCP; https://www.iccp-portal.org/), the U.S. National Comprehensive Cancer Control Program (NCCCP; https://www.cdc.gov/cancer/ncccp/index.htm), and the European Partnership for Action Against Cancer (EPAAC; http://www.epaac.eu/). In the United States, most states and territories recommended opportunistic screening for oral or oropharyngeal cancer. However, District of Columbia, New Jersey, Guam, Northern Marianas Islands, and American Samoa advised targeted screening for high-risk group. Only the plans of North Dakota and West Virginia recommended self-examination. In Australia, only the Victorian region recommended opportunistic screening for oral or oropharyngeal cancer, and in the United Kingdom, this recommendation was followed by Northern Ireland.

The main strategies of national cancer control programs for early detection of oral and oropharyngeal cancer were addressed by 46% of the self-governing political entities and similar entities included. Of these, 29% recommended opportunistic examination, 14.5% suggested screening of high-risk individuals, and 2.4% advocated population-based screening. Only Cuba and two countries in Southeast Asia (India and Myanmar) indicated population-based screening (Figure 4). Regardless of the type of screening, visual/oral examination was the preferred method. The recommended time interval between assessments ranged from 1 to 5 years. Regarding age, Cuba suggested the youngest age range (screening those over 15 years old) and Bhutan the oldest (over 40 years old). Providing education for the general population on oral self-examination was recommended by seven self-governing political entities and similar entities (5.6%). Of these, five (Bangladesh, Panama, Sudan, and the U.S. states of North Dakota and West Virginia) also indicate opportunistic examination for early detection, and the other two suggested population-based screening (India) and screening of high-risk individuals (Sri Lanka). Telemedicine to support oral and oropharyngeal cancer prevention strategies was only mentioned by Chile.

Notably, the cancer plans from Virginia (United States), Hungary, Palau, Thailand, and Suriname advocated training physicians and dentists to detect oral cancer lesions. India recommended screening by physicians, dentists, and community health workers, whereas the Maldives indicated screening by doctors, dentists, community health workers and nurses. In the case of state do Arkansas (United States), the recommendation extended to doctors, dentists, nurses, and dental hygienists.

## Discussion

Despite WHO recommendations for the formulation or adjustment of oral health promotion and prevention policies and strategies, including for oral and oropharyngeal cancer ^15^, the findings of the present scoping review indicated that a significant number of WHO Member States do not include oral and oropharyngeal cancer prevention strategies in their cancer plans and NCD plans on websites with potentially relevant documents for action against cancer: ICCP, NCCCP, and EPAAC.

Evidently, some plans may not have been included in this review due to unavailability on the platforms created for consultation. Search for non-WHO Members was unsuccessful on the websites used for data collection. We performed an additional search on government websites to identify national policies or strategies concerning oral and oropharyngeal cancer, but the lack of standardization across Health Ministries and Cancer Institutes websites in different countries and the difficulties in translating various languages resulted in an ineffective and inconsistent search process. Consequently, the present findings may not fully represent oral and oropharyngeal cancer policies or the acknowledgment of tobacco use, alcohol consumption, sun exposure, HPV infection, and unhealthy diet as risk factors, as other documents may exist in countries whose plans were not included.

Regrettably, many plans were excluded as they did not specifically address oral and oropharyngeal cancer-associated risk factors. Some plans discussed tobacco control strategies to prevent lung cancer and other diseases but did not emphasize the risk for oral and oropharyngeal cancer. Similarly, although many plans cited means to combat excessive alcohol consumption, HPV infection, and unprotected sun exposure as preventive measures against, respectively, liver, cervical, and skin cancer, they did not link these factors to oral and oropharyngeal cancer. Despite the well-established association between tobacco use and an increased risk of oral cancer, only 79 (63.7%) self-governing political entities and similar entities included here explicitly reported this link in their publicly accessible cancer plans. This does not imply that the remaining 45 (36.3%) self-governing political entities, or the other countries excluded from the review sample due to the absence of specific oral and oropharyngeal cancer references, are unaware of tobacco as a health risk factor or lacked preventive measures. Rather, it indicates that oral and oropharyngeal cancer-specific information was not included in the available documents despite covering preventive measures against tobacco use.

Among those indicating oral and oropharyngeal cancer prevention strategies, most focus on primary prevention and less than half address secondary prevention. Moreover, the plans showed a significant heterogeneity in the strategies presented.

Of the plans included in this review, only 19.1% explicitly discuss the three main well-defined risk factors associated with oral and oropharyngeal cancer, i.e., tobacco, alcohol, and HPV. Notably, only the cancer plan of Illinois cited all the well-established oral and oropharyngeal cancer risk factors, including tobacco, alcohol, HPV, diet, and lip sun exposure.

Several plans (21%) focused solely on HPV, followed closely by those that only addressed tobacco (20%). This emphasis on HPV probably stems from the progressive increase trend in HPV-related oropharyngeal cancer over the past two decades in several countries ^16^ ^,^ ^17^, particularly among youth and men ^18^. Immunization plays a pivotal role in preventing a significant percentage of morbidity, disability, and deaths associated with cancer-causing infectious agents ^19^. Including HPV vaccination in the WHO Expanded Program on Immunization (EPI) has led to significant advancements in cancer prevention (Box 1). While some plans outline strategies to expand HPV vaccination programs to reduce cervical cancer incidence, limited attention is given to raising awareness and knowledge about the risk of HPV infection for oropharyngeal cancer. Remarkably, among the ten countries with the highest oropharyngeal cancer incidence, as selected by the age-standardized rate indicator for both genders on GLOBOCAN 2020 (Denmark, France, Romania, Belarus, Cuba, Hungary, Republic of Moldova, Slovakia, Slovenia and Australia) ^20^, seven overlooked HPV infection as a crucial risk for oral and oropharyngeal cancer in their plans, highlighting a significant gap in addressing the disease burden.

Tobacco use (in any form) remains the leading preventable cause for oral and oropharyngeal cancer ^21^ ^,^ ^22^. In 2003, WHO Member States adopted the first public global health treaty - the WHO Framework Convention on Tobacco Control -, adopting preventive measures against the globalization of the tobacco epidemic ^23^. The IARC handbooks program’s first evaluation of oral cancer prevention found that tobacco smoking and alcohol consumption are the main drivers of oral cancer in most countries, with smokeless tobacco use and chewing of areca nut products standing as the leading causes in others, especially in South and Southeast Asia and the Western Pacific Islands ^13^. Plans from self-governing political entities and similar entities in Southeast Asia were the ones that more addressed oral and oropharyngeal cancer risk factors and strategies, which is in line with the high incidence of the disease in this region ^24^ ^,^ ^25^. In the Western Pacific region, however, only 24.2% self-governing political entities and similar entities presented risk factors and/or preventive strategies for oral and oropharyngeal cancer.

Tobacco control remains a critical public health priority worldwide, with numerous strategies being developed and implemented to combat the detrimental effects of smoking (Box 1). We observed interesting and different measures, some of them leveraging the impact of social media to advocate for healthy lifestyle paradigms via partnerships with influential figures, including athletes and renowned artists. This approach effectively targets youth and children by using platforms like TikTok, Facebook, and Instagram, encouraging them to adopt healthier behaviors and steer clear of smoking. Another promising approach involves using technology to develop a phone application to assist individuals in quitting smoking by providing personalized support, resources, and mechanisms, thus empowering users on their journey toward tobacco cessation. Moreover, implementing public health system strategies plays a crucial role in ensuring widespread access to evidence-based cessation programs by standardizing tobacco screening, referrals, and interventions within healthcare settings thereby enhancing the likelihood of timely and effective support for individuals, especially the vulnerable.

Most of the reviewed plans fail to mention electronic nicotine delivery systems (ENDS) and their potential deleterious effects ^26^. Some documents, particularly those from U.S. states, highlight the existing evidence on ENDS use not being risk-free. Conversely, the New Zealand plan advocates regulating vaping products as a means of supporting smokers in transitioning to a less harmful alternative while also safeguarding children and youth against access to and use of these products.

The combination of tobacco use and alcohol intake increases the risk of oral and oropharyngeal cancer cancer development ^27^. Surprisingly, only 35.5% of the plans focused on halting alcohol consumption to prevent oral cancer. Endorsement of WHO’s Global Alcohol Action Plan 2022-2030 emphasizes the importance of prioritizing the reduction of harmful alcohol use in public health efforts targeting oral cancer prevention ^28^. While public health initiatives have successfully decreased tobacco and alcohol prevalence thus contributing to a decline in oral cancer incidence, these achievements are likely a result of comprehensive campaigns focusing on physical activity and nutrition, albeit not explicitly directed toward oral cancer ^29^. Promoting awareness on an individual and community-based level of the harmful effects of alcohol and its association with cancer is generally effective in discouraging consumption ^30^. Based on data collected from the plans, targeted communication campaigns, social media posting, and school health programs emerge as viable measures for raising awareness and addressing alcohol consumption. Additionally, the need to strengthen intersectoral collaboration among agencies involved in alcohol intake control, including health, education, law enforcement, and policy-making, was emphasized to ensure a comprehensive and impactful approach to effectively combat alcohol consumption.

Our findings suggest that primary prevention strategies are widely addressed by the countries that cite oral and oropharyngeal cancer probably due to WHO guidelines and the recognition of the implications these risk factors have for various cancer types and other illnesses.

In embracing technological innovations, telehealth emerges as a transformative tool to enhance the effectiveness of preventive measures. Several plans, especially those from U.S. states, highlighted strategies like expanding mass media campaigns, youth-focused community efforts, HPV vaccine reminders, and electronic screening for behavioral counseling in healthcare services. Moreover, telehealth was recognized as a valuable tool for promoting smoking cessation quitlines and widely disseminating early cancer warning signs. This can be particularly relevant in places with limited healthcare access where technological innovation makes preventive strategies more accessible, benefiting a broader population ^31^ ^,^ ^32^.

In addition to combating oral and oropharyngeal cancer risk factors, secondary prevention strategies could improve patient prognosis by early detection of the disease and appropriate treatment provision. Cancer screening, a key aspect of secondary prevention, offers two primary benefits: reduction of mortality and morbidity ^33^ ^,^ ^34^. In oral cancer prevention, screening also aims to identify individuals with oral potentially malignant disorders, a group of disorders with an increased risk for oral cancer ^7^ ^,^ ^34^. Clinical oral examination is the standard screening method, and evidence suggests that this low-cost approach effectively decreases oral and oropharyngeal cancer mortality in high-risk populations ^13^ ^,^ ^35^. Despite WHO recommendations, oral cancer screening for high-risk groups is not classified as a “Best-Buy” intervention, which indicates high-priority interventions ^12^.

Results revealed that most countries implementing secondary prevention strategies employ opportunistic examination, including nations with a high oral and oropharyngeal cancer rate (e.g., Australia, Hungary, and Bangladesh), the effectiveness of which during regular dental visits is hindered as individuals with risk factors for oral and oropharyngeal cancer are less likely to seek dental care leading to a phenomenon known as “inverse screening law” ^36^ ^,^ ^37^. Consequently, opportunistic examination for early detection of oral cancer often ends up targeting individuals who are at a lower risk for the disease. Despite WHO recommendations, screening for high-risk groups was suggested only by 14.5% of the plans citing preventive measures for oral and oropharyngeal cancer. Additionally, 29% of self-governing political entities and similar entities recommend opportunistic examination and 2.4% population-based screening. Notably, 5.6% of the plans described self-examination alongside other preventive measures.

In Kerala, India, the only randomized clinical trial conducted on oral cancer screening did not initially provide evidence for mortality reduction among the general population; however, a reanalysis conducted in 2021 revealed the effectiveness of oral cancer screening when specifically aiming at high-risk individuals. Additionally, visual inspection performed by trained health professionals proved to be an effective method for early detection. These findings highlight the importance of screening high-risk populations and ensuring adequate training for healthcare providers to conduct visual inspections for early detection of oral cancer ^10^ ^,^ ^11^ ^,^ ^38^. According to Bouvard et al. ^13^, using risk-based models for screening could be an appropriate approach for communities with high oral cancer incidence, despite acknowledging the programmatic challenges in selecting participants. Plans of several countries endorsed this method, including Bhutan, Finland, Malaysia, Marshall Islands, Micronesia, Nigeria, Palau, Seychelles, Sri Lanka, Suriname, Thailand, Timor-Leste, Zambia, as well as U.S. states and territories such as District of Columbia, New Jersey, Guam, Northern Marianas Islands, and American Samoa.

Despite insufficient evidence supporting the effectiveness of population-based screening for oral cancer, India and Cuba, among the ten countries with the highest oral and oropharyngeal cancer incidence ^20^, endorse this strategy. Myanmar also recommends this approach despite present low incidence of the disease. This highlights the varied stances taken by countries with differing incidence rates in advocating for population-based screening.

Oral self-examination has been proposed as a simple, noninvasive procedure to facilitate early detection of oral cancer as it does not require a healthcare professional appointment. However, accurately identifying the absence of potentially malignant and malignant oral lesions is a challenge in this approach. Evidence supporting oral self-examination and remote screening is limited ^3^ ^,^ ^39^.

The negative impact of cancer on a country’s health and development cannot be ignored. All governments are responsible for fulfilling the United Nations Resolution Goals - 2030 Agenda for Sustainable Development and achieving the best possible results in the fight against cancer. Implementing the necessary measures requires policy formulation based on available data, the appropriate mobilization and allocation of resources, active participation of all stakeholders and, above all, the government’s commitment to fostering education, equity in health, and initiatives to improve access and ensure comprehensive care in areas of greater vulnerability. As reinforced by the reviewed Plans, significant advances have been made in cancer prevention such as tobacco control programs and inclusion of HPV vaccination in the EPI. However, the focus on primary prevention suggests an overemphasis on individual accountability for risky behaviors. While promoting awareness and behavior change among individuals is crucial, it is equally important that governments take responsibility for improving the provision and quality of healthcare services, especially regarding early diagnosis and treatment, to maximize impact on reducing cancer mortality. Lack of balance between these two approaches can limit oral and oropharyngeal cancer prevention effectiveness.

Finally, results show that oral and oropharyngeal cancer prevention strategies were absent from cancer or NCD plans available on the consulted platforms for numerous countries. In cases where countries did recommend specific strategies, we observed significant diversity in both primary and secondary prevention actions with some critical points, such as the lack of correlation between oral and oropharyngeal cancer and the main risk factors. The impact of implementing oral and oropharyngeal cancer prevention strategies must be studied and reported over the long term, particularly in correlation with incidence and mortality data. A recent study by Martínez-Ramírez et al. ^40^ point out limited implementation of oral cancer control plans as a major barrier to early diagnosis and management in Latin America and the Caribbean. It is crucial to assess the effectiveness of prevention measures in tackling the specific challenges posed by oral and oropharyngeal cancer and to monitor their influence on disease trends and outcomes. Long-term studies could provide valuable insights into the success of these strategies, contributing to refine global efforts in combatting oral and oropharyngeal cancer.

## Conclusion

Our scoping review highlights that a significant number of WHO Member States do not include prevention strategies specifically tackling oral and oropharyngeal cancer in their cancer and NCD plans available on key organization websites. This should not imply an absence of an oral and oropharyngeal cancer policy in these countries, as other documents may exist. Plans indicating actions for oral and oropharyngeal cancer prevention focused significantly on primary prevention and a great variability in the presented strategies. The growing burden of this disease in many countries underlines the urgent need for enhanced public awareness and early detection efforts. Collaboration among healthcare providers, policymakers, and community stakeholders is crucial for implementing effective strategies, and our results can contribute to developing and improving cancer plans to combat oral and oropharyngeal cancer.
